# A Case of Suspected Radiation Recall Pneumonitis After a COVID-19 Infection

**DOI:** 10.7759/cureus.13688

**Published:** 2021-03-04

**Authors:** Hiromasa Kurosaki, Nobuko Utsumi, Kosei Miura

**Affiliations:** 1 Department of Radiation Therapy, JCHO Tokyo Shinjuku Medical Center, Tokyo, JPN; 2 Department of Radiation Oncology and Proton Medical Research Center, University of Tsukuba, Tsukuba, JPN

**Keywords:** radiation recall reaction, covid-19, radiotherapy, lung cancer

## Abstract

We present the case of a 78-year-old woman who received definitive radiation therapy for small cell lung cancer three and a half years ago. She was asymptomatic when she tested positive for coronavirus disease 2019 (COVID-19). She then developed a rapid decline in respiratory status on day seven. Chest radiograph revealed a strong shadow at the prior irradiation site. Radiation recall reactions are usually caused by drug administration, but in this case, it was suspected to be caused by COVID-19.

## Introduction

The novel coronavirus disease 2019 (COVID-19) outbreak occurred in Wuhan City, Hubei Province, China in December 2019. As of early February 2021, there have been over 100 million infectious cases with one million deaths worldwide.

COVID-19 significantly impacts radiation therapy, particularly promoting radiation therapy postponement and fractionated irradiation [1]. However, the pathophysiology and other involved factors are unknown. We report a case of suspected radiation recall pneumonitis three and a half years after definitive radiotherapy for lung cancer.

## Case presentation

**Figure 1:**
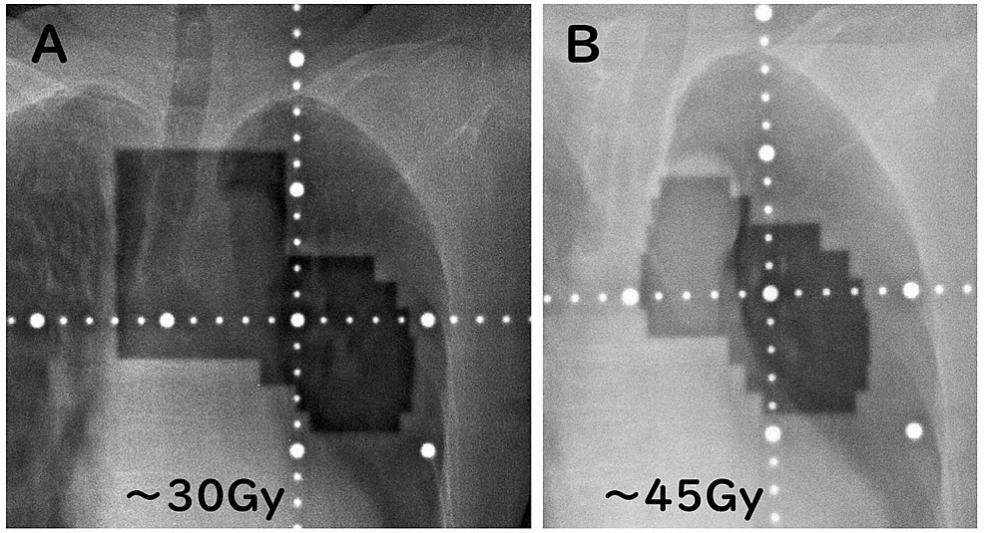
Portal images for small cell lung cancer. (A) 30 Gy/20 fractions; (B) 15 Gy/10 fractions.

**Figure 2:**
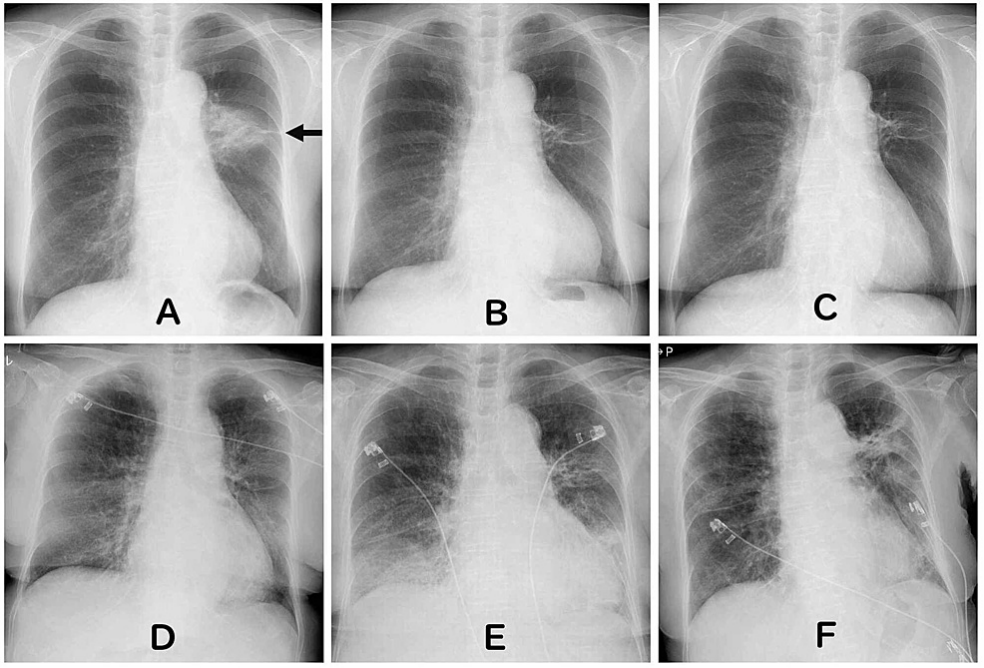
X-p image. (A) X-p five months after curative radiation therapy: radiation pneumonia consistent with the irradiation field was observed. (B) X-p 3.5 years after the curative radiation therapy. This is two weeks before SARS-CoV-2 positivity: radiation-induced pulmonary fibrosis was observed. (C) X-p one day prior to SARS-CoV-2 positivity: no change from two weeks prior. (D) X-p on the day of rapid deterioration of respiratory condition observation (day seven): appearance of ground-glass shadow showing dilated bronchi inside. (E) X-p two days later: a shadow consistent with the irradiation was observed (day nine). (F) X-p 46 days after SARS-CoV-2 positivity. X-p, chest radiograph; SARS-CoV-2, severe acute respiratory syndrome coronavirus 2

A 78-year-old woman was diagnosed with small cell lung cancer three and a half years ago and underwent definitive chemoradiotherapy. Radiation therapy was performed via accelerated hyperfractionation, followed by 30 Gray (Gy)/20 fractions in two anterior-posterior opposing phyla, followed by 15 Gy/10 fractions. Figure 1 shows the portal images at the time of the treatment. Radiation pneumonitis was not observed at the end of the radiation therapy. However, radiation pneumonitis was confirmed on the chest radiograph (X-p) five months after the completion of irradiation (Figure 2A).

**Figure 3:**
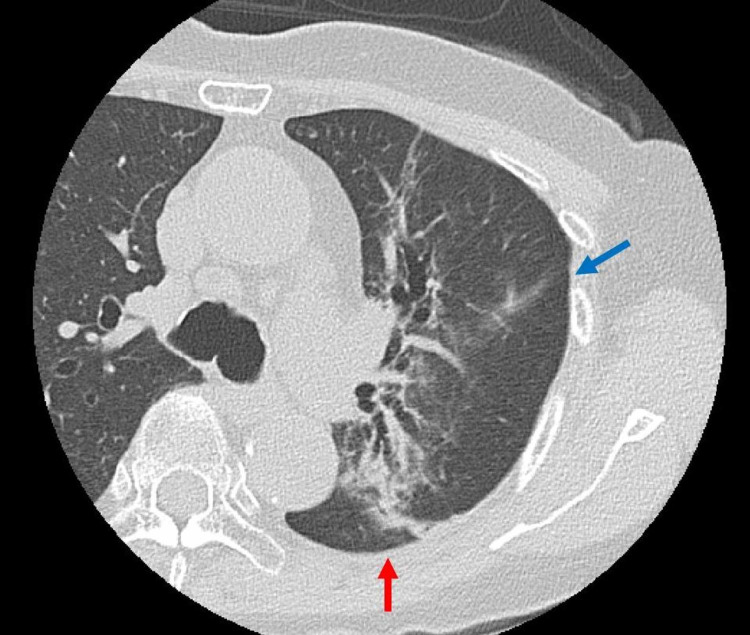
CT image 1.5 months prior to COVID-19 infection. The red arrow shows radiation fibrosis and the blue arrow shows interlobar fissure. CT, computed tomography; COVID-19, coronavirus disease 2019

Brain metastasis was detected about two years after radical radiation. Stereotactic radiotherapy and surgery were performed. Computed tomography (CT) scan was performed three years and four months after the radical radiation (Figure 3). Recurrence in the trunk was absent during this period.

However, three years and five months after radical radiation in April 2020, because of the presence of multiple brain metastases, whole-brain irradiation was performed. Figure 2B shows the X-p scan at the start of whole-brain irradiation.

Whole-brain irradiation was performed in the hospital. During the time of her admission, multiple COVID-19 cases occurred in the hospital ward. Therefore, the patient was tested for COVID-19 despite being asymptomatic. A polymerase chain reaction (PCR) test was performed on the nasopharyngeal swab, and severe acute respiratory syndrome coronavirus 2 (SARS-CoV-2) positivity was confirmed. Figure 2C shows the result of the X-p performed the day before the PCR test. Whole-brain irradiation of 30 Gy/10 fractions was planned; however, the treatment was terminated upon reaching 18 Gy.

Hydroxychloroquine treatment was initiated two days after she was tested positive. Although the patient was asymptomatic, a decrease in percutaneous oxygen saturation (SpO_2_) to 94% was observed from day four.

On day seven, a sharp decline in SpO_2_ to 74% was observed. However, she was still asymptomatic. Her respiratory condition rapidly deteriorated with partial pressure of oxygen of 47.6 mmHg and partial pressure of carbon dioxide in arterial blood of 29.9 mmHg while on 4 L of oxygen. X-p from the same day is shown in Figure 2D. Dyspnea was ameliorated via administration of oxygen and ceftriaxone/heparin. On day nine, a strong shadow was observed within the irradiation field (Figure 2E). A strong shadow was also observed on the X-p on day 46, consistent with the irradiation field (Figure 2F).

Negative PCR test results were confirmed on the nasopharyngeal swab on day 53. The patient died of primary cancer three months after the confirmation of a positive test result; however, CT and autopsy were not performed owing to COVID-19 positivity.

## Discussion

A radiation recall reaction is an inflammatory reaction localized within the previous irradiation site. It is caused by the administration of various drugs post irradiation. Acute dermatitis that develops a few days to a few weeks after drug administration is the most commonly reported symptom. Mucositis and interstitial pneumonia may develop simultaneously or individually [2].

Details of the pathogenic mechanism of irradiation recall reactions have not been elucidated [2]. The first mechanism claims that cytotoxic drugs trigger inflammatory reactions among the cells remaining in the irradiation field after radiation therapy. Second, genetic mutations in the cells remaining after radiation therapy and a decrease in the number of tissue stem cells allow for recall reactions. It was previously speculated that these factors induce resistance to additional chemotherapy and cause inflammatory responses. Notably, it is not only caused by non-cytotoxic drugs such as tamoxifen but also by recently developed immune checkpoint inhibitors [3-5]. Therefore, immunity is also considered to be an influencing factor, but the mechanism is still unknown.

In this patient, radiation pneumonia was confirmed after irradiation, while radiation fibrosis shadow was confirmed before COVID-19 infection (radiation pneumonitis typically occurs within six months after radiotherapy). A shadow in the radiation field was observed two days after the onset of COVID-19 (day nine, Figure 2E). The shadow matching the irradiation field was likely due to COVID-19 and was radiation fibrosis before. Only ceftriaxone and heparin were administered, and there have been no reports of radiation recall reactions with these drugs.

On the other hand, all radiation recall reactions reported so far have been drug-induced, and based on our research, there has been no report of a case caused by an infectious disease. It is known that COVID-19 causes immune system reactions such as cytokine storms. This may have induced a radiation recall reaction, as observed in this case. In addition, a decrease in SpO_2_ was observed three days before the onset, and a happy hypoxia may have gradually developed. Happy hypoxia, which is a characteristic of COVID-19, has been hypothesized to be caused by various factors, including angiotensin-converting enzyme 2 [6,7]. It causes gradual lung damage, which may lead to radiation recall pneumonitis.

Moreover, the pathology report had incidental findings of early-stage COVID-19 when surgery performed for lung cancer included edema, prominent proteinaceous exudates, vascular congestion, and inflammatory clusters with fibrinoid material [8,9]. This could indicate with some possibility that a small amount of SARS-CoV-2 virus was deposited in the alveolae of both lungs and could cause cytokine release, leading to radiation recall pneumonia.

Zeng et al. pointed out a number of similarities of clinical symptoms, laboratory findings, and CT imaging features between COVID-19 pneumonia and radiation pneumonia [10]. The efficacy of COVID-19 treatment is well demonstrated on follow-up CT imaging. If symptoms have improved, less ground-glass opacities are seen [11]. Taking this into account, if ground-glass opacities are still seen on the X-p taken on the 46th day, it is highly likely that radiation recall is indicated rather than COVID-19 infection.

There were cases of COVID-19 infection after radiation therapy in the first wave of COVID-19 in China [10,12,13]. These were all mild cases, and the region of ground-glass patterns differed for each case. If a patient who received chest irradiation is infected with SARS-CoV-2, it is recommended to follow the course of the chest findings.

## Conclusions

We described a case in which radiation recall pneumonitis was suspected after COVID-19 pneumonitis three and a half years post curative radiation therapy. There are still many uncertainties that are poorly understood about COVID-19. If a patient who received chest irradiation is infected with SARS-CoV-2, it is suggested to follow the course of the chest findings.
